# Diagnostic Yield and Safety of Endoscopic Ultrasound Guided Fine Needle Aspiration of Central Mediastinal Lung Masses

**DOI:** 10.1155/2013/150492

**Published:** 2013-05-30

**Authors:** Enrique Vazquez-Sequeiros, Michael J. Levy, Manuel Van Domselaar, Fernando González-Panizo, Jose Ramon Foruny-Olcina, Daniel Boixeda-Miquel, Diego Juzgado-Lucas, Agustin Albillos

**Affiliations:** ^1^Gastroenterology Service, University Hospital Ramon y Cajal, 28801 Madrid, Spain; ^2^Gastroenterology Service, University Hospital Quiron, Madrid, Spain; ^3^Developmental Endoscopy Unit, Gastroenterology Division, Mayo Clinic, Rochester, MN, USA

## Abstract

*Background and Aims*. EUS-FNA is an accurate and safe technique to biopsy mediastinal lymph nodes. However, there are few data pertaining to the role of EUS-FNA to biopsy central lung masses. The aim of the study was to assess the diagnostic yield and safety of EUS-FNA of indeterminate central mediastinal lung masses. *Methods*. Design: Retrospective review of a prospectively maintained database; noncomparative. Setting: Tertiary referral center. From 10/2004 to 12/2010, all patients with a lung mass located within proximity to the esophagus were referred for EUS-FNA. Main Outcome Measurement: EUS-FNA diagnostic accuracy and safety. *Results*. 73 consecutive patients were included. EUS allowed detection in 62 (85%) patients with lack of visualization prohibiting FNA in 11 patients. Among sampled lesions, one patient (1/62 = 1.6%) had a benign lung mass (hamartoma), while the remaining 61 patients (61/62 = 98.4%) had a malignant mass (primary lung cancer: 55/61 = 90%; lung metastasis: 6/61 = 10%). The sensitivity, specificity, and accuracy of EUS-FNA were 96.7%, 100%, and 96.7%, respectively. The sensitivity was 80.8% when considering nonvisualized masses. One patient developed a pneumothorax (1/62 = 1.6%). *Conclusions*. EUS-FNA appears to be an accurate and safe technique for tissue diagnosis of central mediastinal lung masses.

## 1. Background

Lung cancer represents the most common cause of cancer and cancer-related mortality [1]. Treatment strategy largely depends on the tumor stage, with tissue confirmation considered necessary to provide therapy. Pulmonologists and thoracic surgeons have classically relied on bronchoscopy (forceps biopsy of lumen tumor and/or blind transbronchial fine needle aspiration), computed tomography- (CT-) guided fine needle aspiration/biopsy (FNA), mediastinoscopy, or thoracoscopy for diagnosis [2]. 

The technique selected usually depends on local expertise and tumor and nodal location. The accuracy of each of these techniques is limited and each is associated with notable morbidity [2]. The sensitivity of bronchoscopically guided biopsy is poor, as it fails to reach a definitive diagnosis in 20 to 30% of patients [3]. CT-guided FNA provides a diagnostic accuracy of 77% to 95%, but is largely limited to sampling of masses larger than 2 cm in size and may result in pneumothorax in as many as 22%–62% of patients [4–6]. In addition, CT often cannot be performed in lesions abutting or located in close proximity to large mediastinal vessels [7]. While surgical techniques such as mediastinoscopy or thoracoscopy provide excellent diagnostic accuracy in this setting, both must be performed in the operating room under general anesthesia and have been associated with significant procedure-related mortality (as high as 0.4%) [8].

More recently, endoscopic ultrasound guided fine needle aspiration (EUS-FNA) has proven to be an accurate and safe technique to biopsy mediastinal lymph nodes to aid lung cancer staging [9–11]. In addition, limited data suggest that EUS-FNA may also allow imaging and biopsy lung masses and may complement or substitute for other biopsy techniques [12–14]. In recent years, pulmonologists and thoracic surgeons have also employed endobronchial ultrasound (EBUS) to examine and biopsy (EBUS-TBNA) lesions in the anterior mediastinum, with excellent results (accuracy > 80–90%) [11, 15]. Unfortunately, this modern technique is still beginning to spread in many countries and is not available in many institutions. 

*Aim. *The aim of this study was to determine the diagnostic yield and safety of EUS-FNA in patients with a central mediastinal lung mass. 

## 2. Patient and Methods 

### 2.1. Study Design

We conducted a retrospective review of a prospectively maintained database (at 2 tertiary referral centers) to identify consecutive patients with an indeterminate central lung mass who were referred for EUS-FNA. Central mediastinal lung masses were defined as those with the closest margin believe to be in sufficient proximity to the esophageal wall based on pre-EUS-CT or PET-CT to potentially permit transesophageal EUS-FNA. The study includes all consecutive patients evaluated since the first referral in November 2004 until January 2011. Of note, while prior biopsy efforts may have included bronchoscopy and/or CT-guided biopsy, endobronchial ultrasound (EBUS) and EBUS-guided FNA were not available in our institutions at the time of the study. Clinical followup was available for a minimum of 12 months in each patient. The study protocol was approved by the Institutional Review Board, and informed consent was obtained for all procedures. 

In accordance with our standard of clinical practice, this study excludes patients with (1) serious comorbidities precluding EUS exam; (2) patient refusal to participate; (3) prior tissue diagnosis by other means; (4) other target lesions more easily accessible for biopsy (e.g., liver metastasis); (5) coagulation disorder precluding EUS-FNA (platelet count <50,000/*μ*L and/or international normalized ratio INR > 1.5); or (6) prior radiation therapy for lung cancer. 

**Figure 1:**
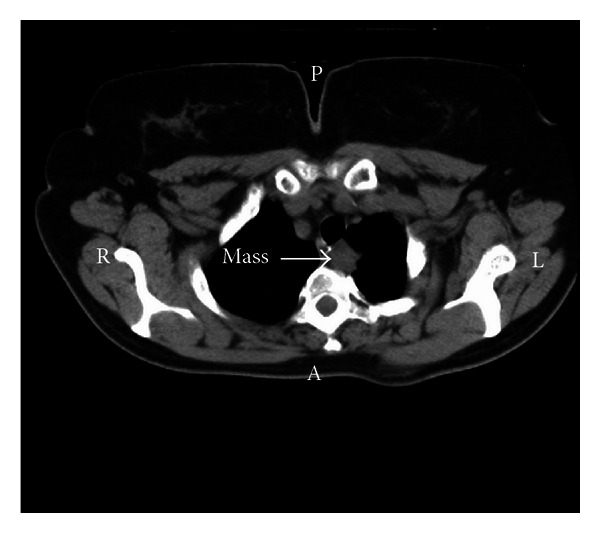
Chest CT scan showing a left upper-lobe lung mass adjacent to the esophagus.

Patient data (demographics, intervention, and followup) were prospectively collected and introduced in a predefined computer database for later review. A chest CT scan and/or a PET/CT scan demonstrating a lung mass adjacent to the esophagus, with no mediastinal lymphadenopathy and/or distant metastases, was obtained in all cases (Figure 1). Bronchoscopy was also performed in all patients.

### 2.2. EUS Exam and FNA

**Figure 2:**
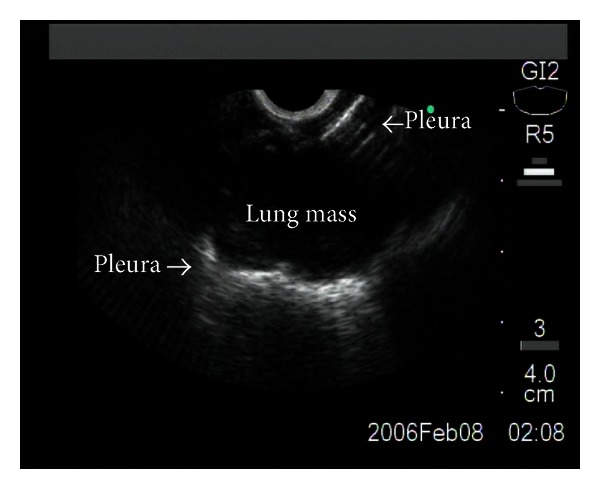
EUS exam of the same patient showing the hypoechogenic mass, 2 cm in size, located in the lung parenchyma.

**Figure 3:**
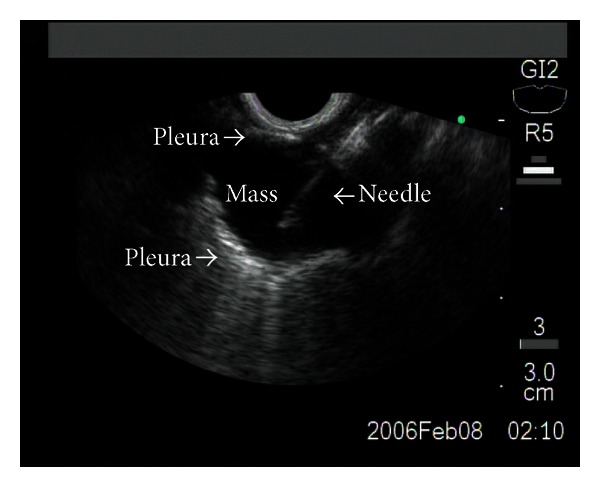
EUS-FNA of the lung mass identified was performed, disclosing a diagnosis of nonsmall-cell lung cancer.

The EUS-FNA exam was performed on an outpatient basis by an endosonographer having performed more than 5000 EUS procedures using a curvilinear array echoendoscope (GF-UCT140-AL5, GF-UCT160-OL5 Olympus 5–10 MHz) and an Aloka (ProSound SSD-*α*5) or C-60 ultrasound processor. Conscious sedation was administered using a combination of midazolam, propofol, and fentanyl or meperidine. The EUS instrument was advanced into the stomach with continuous imaging during withdrawal to exam the liver, left adrenal gland, perigastric region, and posterior mediastinum. A 22-gauge needle with stylet (Echo-Tip; Wilson Cook Medical Inc., Winston-Salem, North Carolina) was inserted in the mass under EUS guidance (Figures 2 and 3). In the preset study, we employed 25 gauge needles for biopsy. Per routine, after withdrawing the needle stylet, five cc of negative pressure was applied while 10 to and from movements were performed within the lesion. During subsequent needle passes, the amount of negative pressure was increased or decreased (range 0–10 cc) if the sample was grossly sparse or bloody appearing, respectively. When necessary, color Doppler US was used to avoid accidental puncture of intervening vascular structures.

The collected material was sprayed onto glass slides and evaluated by an on-site cytopathologist to assess adequacy. EUS-FNA aspirates were obtained until (1) a preliminary on site cytopathologic diagnosis of malignancy was given; or (2) a maximum of 3 passes had been performed, and adequate and representative material had been obtained by EUS-FNA means as assessed by the cytopathologist.

The definitive EUS-FNA cytologic diagnosis was based on formal review of all materials (Diff-Quik and Papanicolaou) with a positive test result mandating an interpretation of “positive for malignancy.” All nonpositive interpretations (i.e., negative, atypical, and suspicious interpretations) were considered negative for malignancy. 

As per routine clinical practice, patients were monitored after procedure for a minimum of 90 minutes during which development of complications was assessed. Patients were also evaluated for complications at standard clinical followup that is performed within 7 days after the procedure, during which a physical exam and chest X-ray were routinely obtained. Based on the patient status at initial followup, additional phone or in-person followup was conducted to identify any late complications. 

### 2.3. Gold Standard for Lung Mass Diagnosis

 The gold standard was based on strict cytohistologic correlation and a minimum 12-month followup [16]. A patient was considered to have malignancy if there was (1) cytologic and/or histologic evidence of malignancy based on material obtained via (a) EUS FNA, (b) surgical pathology (for those patients receiving surgical resection or mediastinoscopy or thoracoscopy), (c) percutaneous biopsy, or (d) autopsy; or (2) clinical course (≥12 months) suggesting malignancy based on presence of: (a) new radiographic abnormality including (i) regional or distant mass (hepatic, pulmonary, or bone), (ii) mass infiltrating large blood vessels, or (iii) malignant appearing lymphadenopathy with positive positron emission tomography imaging; or (b) cancer-related mortality. Designation of a lesion as benign required at least 12 months of followup and absence of any of the above criteria and/or follow-up imaging demonstrating complete resolution of the abnormality.

### 2.4. Statistical Analysis

A commercially available statistical software package (JMP 7.0.2 SAS Institute Inc., North Carolina, USA) was employed for statistical analysis. Descriptive analysis of data is presented in the paper as follows: (a) discrete variables, percentage and 95% confidence interval; (b) continuous variables, mean ± standard deviation (or median/interquartile range) and range [17]. The performance characteristics (sensitivity, specificity, and overall diagnostic accuracy) of EUS-FNA in this cohort of patients with a centrally located lung mass were calculated as previously described [17].

## 3. Results 

From November 2004 to January 2011, 73 patients with a central mediastinal lung mass were referred for possible EUS-FNA. EUS allowed detection in 62 (85%) patients with lack of visualization prohibiting FNA in 11 patients. CT revealed that in 9 of the 11 patients the mass was located more than 1 cm distant to the cervical/upper esophagus potentially contributing to the failed EUS detection. In the other 2 patients, the lung mass was located in the mid esophagus, but >2 cm away from the esophageal wall, likely negatively impacting EUS detection. 

For the 62 patients in whom EUS identification was possible, there was a male preponderance (male/female: 48/14 = 78%/22%), with a mean age of 68.0 ± 11.5 years (range 61–82). The lung masses were located in the vicinity of the cervical/upper esophagus (*n* = 19; each <1 cm from esophagus) or mid esophagus (*n* = 43 patients; each <2 cm from the esophagus). The lung masses measured a median long axis of 26 mm (range 12–65 mm), and the median number of EUS-FNA passes performed was 1 (range 1–3). 

**Figure 4:**
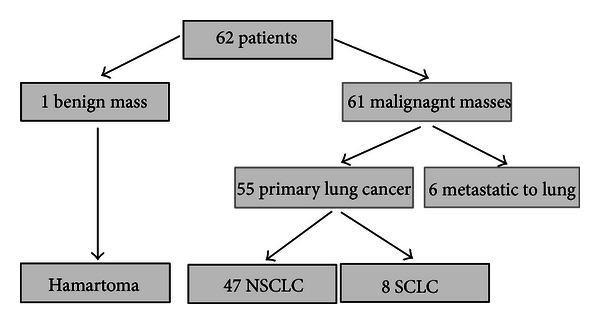
Final diagnosis of the 62 patients in whom EUS detection of the lung mass was possible. *Nonsmall-cell lung cancer (NSCLC); small-cell lung cancer (SCLC).

Based on the gold standard diagnosis, one of the visualized masses (1/62 = 1.6%; 95% CI: 0–9%) was ultimately diagnosed as a benign lesion (hamartoma), while the remaining 61 patients had a malignant mass (61/62 = 98.4%; 95% CI: 90–100%). Fifty-five malignant masses represented primary lung cancers (55/61 = 90%; 95% CI: 80–96%), while 6 were ultimately diagnosed as a lung metastasis (6/61 = 10%; 95% CI: 4–20%). Among the subgroup of primary lung cancers (*n* = 55), 47 were nonsmall-cell lung cancers (NSCLC), and 8 represented small-cell lung cancer (SCLC) (Figure 4).

An adequate EUS-FNA sample was obtained in all but one patient (61/62 = 98.4%; 95% CI: 93–100%), yielding the correct diagnosis in 60 patients (diagnostic accuracy: 60/62 = 96.7%; 95% CI: 88–99). The two false negative EUS-FNA results were obtained in patients with NSCLC, in which the EUS-FNA specimen contained only benign cells (*n* = 1) or no cytologic material was obtained (*n* = 1). The tumor sizes for these later two patients were 12 and 25 mm, respectively. Therefore, the sensitivity of EUS-FNA among all 73 patients was 80.8% (59/73; 95% CI: 70–89%) compared to 96.7% (59/61 = 96.7%; 95% CI: 88–99%) for the cohort in whom EUS lung mass detection was possible. 

One patient (1/62 = 1.6%; 95% CI: 0–9%) with a NSCLC developed a large tension pneumothorax within 24 hours of EUS-FNA-requiring chest tube placement and 24-hour intensive care unit observation. The patient fully recovered and was discharged from the hospital 7 days later. This complication was not recognized during the EUS-FNA procedure and was diagnosed due to patient dyspnea after release from the endoscopy unit. No other complications developed. 

## 4. Discussion

The key to the evaluation and management of a lung mass is determining whether it represents a malignant or benign [18]. Standard techniques for obtaining a tissue diagnosis such as bronchoscopy or CT-guided biopsy yield a false negative rate of approximately 30% and risk pneumothorax [3–6]. More recently, introduced techniques such as EUS-FNA and EBUS-TBNA provide enhanced diagnostic sensitivity [9–16]. EUS-FNA is now routinely performed to evaluate indeterminate mediastinal structures and to enhance NSCLC staging largely through the targeted inspection of posterior mediastinal nodal stations (left lower paratracheal, aortopulmonary window, subcarinal, lower paraesophageal, and inferior pulmonary ligament) [19–30], providing a sensitivity and specificity of 81–97% and 83–100%, respectively [31]. EUS-FNA may be useful as the first diagnostic test in patients with suspected lung cancer or following negative bronchoscopy and computerized tomography [32]. EUS-FNA and EBUS-TBNA are now regarded as complementary staging modalities that combine to provide a diagnostic accuracy of 91% similar to surgical techniques [33–36]. Some suggest that these techniques may substitute for surgical diagnosis and staging of lung cancer patients [33–36].

Although EUS-FNA has been shown to be extremely useful for evaluation of mediastinal lymph nodes and masses in a minimally invasive manner, there are a paucity of data regarding the role of EUS-FNA to cytohistologically diagnose lung parenchyma masses. Varadarajulu et al. [12] conducted a retrospective study including 18 patients undergoing EUS-FNA of a lung mass abutting the esophageal wall. They reported a diagnostic yield of 100% without complications. Annema et al. [13] conducted a prospective and controlled study to assess the feasibility and diagnostic yield of EUS-FNA for diagnosis of centrally located lung tumors following a nondiagnostic bronchoscopy. EUS-FNA provided a diagnosis of malignancy in 31 of the 32 patients (97%) [13]. Hernandez et al. [14] described their retrospective experience in which EUS-FNA of centrally located primary lung cancers was diagnosed in all 17 patients evaluated. 

Our data represent the largest study to date and support experience from other research groups. EUS-FNA provided the correct diagnosis in 96.7% of patients, and further diagnostic interventions (mediastinoscopy) were only required in two cases. Those 2 patients that could not be diagnosed by EUS-FNA corresponded to (1) one patient with a lung mass in which EUS-FNA obtained an inadequate/necrotic and nondiagnostic material; (2) one patient with a malignant lung mass (primary) and a false negative EUS-FNA result. Our findings also support the role of EUS-FNA in evaluating lung masses resulting from secondary metastasis. 

A notable limitation of EUS-FNA is the impaired ability to identify and access lung tumors located distant (>2 cm) to the esophagus, which unfortunately comprise a majority of lung cancers. The impaired detection results from the acoustic impedance mismatch at the lung interface, which greatly limits ultrasound transmission. Our study is also limited by the lack of availability of EBUS and EBUS-TBNA in our centers during the study period. This may have resulted in some degree of referral bias that could theoretically impact our study findings. 

To our knowledge, whether EUS-FNA or EBUS-TBNA may be better suited to perform biopsies of lung masses has not been investigated. Tournoy et al. demonstrated in a retrospective noncomparative study including 60 patients (82% with a prior nondiagnostic flexible bronchoscopy), that EBUS-TBNA reaches a sensitivity of 82% and a negative predictive value of 23% when biopsying centrally located lung masses [15]. Unfortunately, the present study was conducted at two institutions, in which EBUS-TBNA was not available at that time. Therefore, direct comparisons between both techniques could not be performed. We believe a combination of EUS-FNA and EBUS-TBNA will probably be the best approach for diagnosis of central mediastinal lung masses. Future studies designed for direct comparison of both techniques are definitively needed. 

In summary, our data support the accuracy and general safety of EUS-FNA for evaluation of central mediastinal lung masses. Further research is needed to determine the role of EUS-FNA relative to EBUS-TBNA as the initial diagnostic test in patients with an indeterminate lung mass.

## Abbreviations

CI: Confidence interval
CT: Computerized tomography
EUS-FNA: Endoscopic ultrasound guided fine needle aspiration
NSCLC: Nonsmall-cell lung cancer
SCLC: Small cell lung cancer.
